# Physical Radiation Enhancement Effects Around Clinically Relevant Clusters of Nanoagents in Biological Systems

**DOI:** 10.1038/s41598-019-44482-y

**Published:** 2019-05-31

**Authors:** B. Villagomez-Bernabe, F. J. Currell

**Affiliations:** 1 School of Mathematics and Physics, Queen’s University, Belfast, BT7 1NN UK; 2 The University of Manchester The Dalton Cumbrian Facility, Westlakes Science & Technology Park, Moor Row, Cumbria, CA24 3HA UK; 3 School of Chemistry, The University of Manchester, Oxford Road, Manchester, M13 9PL UK

**Keywords:** Nanoscale biophysics, Cancer, Nanotechnology in cancer

## Abstract

Here we show that the determining factor for physical radiation enhancement effects for a clinically realistic cluster of heavy-atom bearing nanoparticles is the total number of heavy atoms packed into the cluster. We do this through a multiscale Monte Carlo approach which permits the consideration of radiation transport through clusters of millions of nanoparticles. The finding is in contrast to that predicted when isolated nanoparticles are considered and is a direct consequence of the Auger electrons playing less of a role for clusters compared to isolate nanoparticles. We further show that this result is agnostic to selection of the subcellular region considered to be sensitive to the effects of radiation, provided the inside the cluster of nanoparticles is not considered to be biologically active.

## Introduction

Nanoparticles with a core containing heavy-atoms and (usually) a coating of lower average atomic number enhance effects of radiation during cancer therapy and have recently entered into clinical trials^1^. Such nanoparticles are almost ubiquitously found clustered together in cells. Whilst in some cases there might be coating-dependent biologically based radiosensitization, a common feature to all such nanoparticles is physical dose enhancement able to act locally to produce large biological effects^2–8^. Although studies involving clusters of nanoparticles have begun to appear in the literature, these have either involved non-clinical beams^9^ or for periodic (and hence clinically unrealistic) arrays of nanoparticles^10^. Using a multi-scale Monte Carlo calculation formalism, we present calculations of physical enhancement due to clusters of many nanoparticles (millions in some cases), i.e. the form in which they are commonly observed.

Heavy atoms can be introduced into tumours either as nanoparticles or within small molecules. In order to study the physical enhancement effects, clusters comprised of three exemplar nanoagents were chosen - auranofin (a small molecule containing a gold atom), a 1 nm radius core gold nanoparticle and a 25 nm radius core gold nanoparticle (AuNP). Both nanoparticles had a 1 nm thick citrate coating although this could equally well have been some other coating made from low atomic number elements.

**Figure 1:**
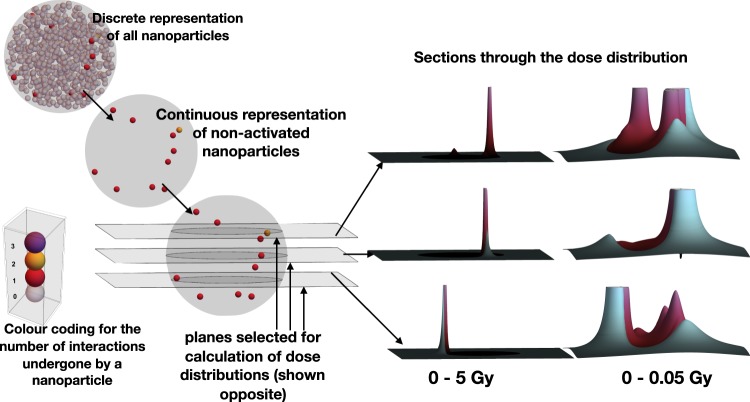
Overview the simulation process, in this case, concerning a spherical, 500 nm radius cluster of 25 nm radius gold nanoparticles (10% volumetric fill factor), irradiated to 1 Gy (dose to surrounding water) with a 6 MeV clinical radiation beam. A nanoparticle cluster is generated stochastically, as is the number of interactions each nanoparticle has with the radiation. The few nanoparticles undergoing interactions remain discretely represented while the remainder are transformed into a continuous representation which preserves the average gold density distribution (see methods for details). To determine the dose distribution at any point, the dose distributions due to the activated nanoparticles are summed in a way which takes account of the attenuation due to the non-activated nanoparticles. This (slightly) unusual cluster was chosen for illustration purposes because it contains a nanoparticle undergoing two activation events. Only about 1/10^th^ of such clusters would show this property. For the particular cluster illustrated above, the dose distribution through 3 planes passing through the cluster are illustrated. ‘Plum’ colours have been used to represent dose deposited within the cluster and ‘blues’ to represent dose deposited outside.

Dose distributions for clusters of nanoparticles were calculated as shown in Fig. 1 under clinically meaningful conditions. By comparing the results for the different nanoagents, new insights into the relative magnitude of physical radiation enhancement effects in biological systems have been deduced. Using this approach and considering large numbers of randomly generated clusters we show that the dominant factor in determining radiation dose enhancement is simply the total number of heavy atoms present. Furthermore, the very large dose enhancements found near small nanoparticles confers no practical advantage and is outweighed by their less efficient packing unless the internal volume of the nanoparticle cluster is considered to be biologically active.

## Results

The Calculations presented start with determination of the radial dose distribution for a single isolated nanoagent and proceed through determination of a series of intermediate quantities to result in predictions about the comparative effect placement and packing have on the physical radiation enhancement effects. Table 1 gives definitions for the key quantities involved and acts as a kind of glossary for the reader.

**Table 1:** Definition of terms used in this paper.

Symbol	Dimensions	Explanation
$L(\overrightarrow{r})$	Dimensionless	Local energy enhancement, i.e. the ratio of the energy deposited due to the nanoparticles to that which would be deposited if no nanoparticles were present, both considered at a point in space $\overrightarrow{r}$
〈*L*〉	Dimensionless	average local energy enhancement, i.e. the average of $L(\overrightarrow{r})$ taken over a sphere of radius *r* centred on the centre of the cluster
*S*_1_(*r*)Δ*r*	nm^3^	Average energy deposited in a thin spherical shell of radius *r* and thickness Δ*r* for a 1 Gy dose applied uniformly (i.e. in the absence of nanoparticles) or equivalently the volume of this shell multiplied by 〈*L*〉.
*S*_1_(*r*)Δ*r*	nm^3^	Average of the square of the energy deposited in a thin spherical shell of radius *r* and thickness Δ*r* for a 1 Gy^2^ applied uniformly (i.e. in the absence of nanoparticles).
*I*_1_	nm^3^	The total additional dose deposited in the sensitive volume due to a single cluster embedded in a 1 Gy background dose.
*I*_2_	nm^3^	The total additional dose-squared deposited in the sensitive volume due to a single cluster embedded in a 1 Gy background dose.

### Radial dose distributions for single nanoparticles

**Figure 2:**
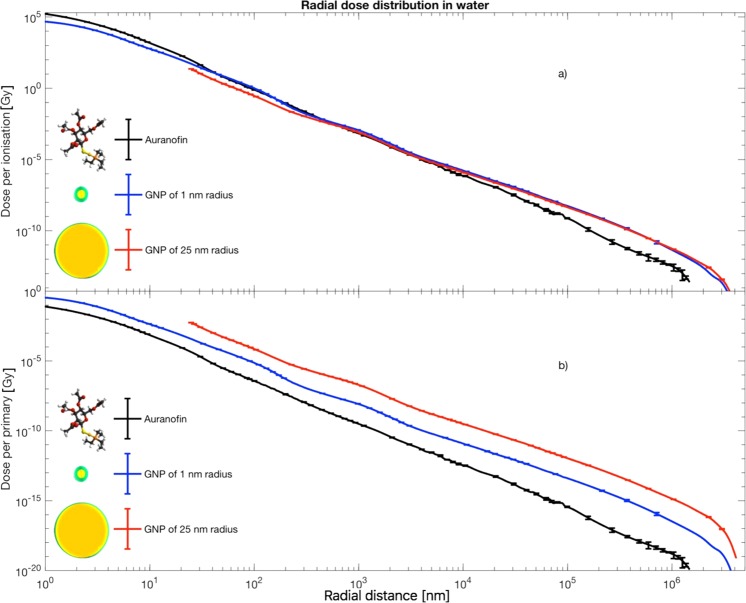
Simulated radial dose distributions (RDDs) of the three candidate nanoagents under consideration when irradiated with 6 MeV linac radiation at a depth of 3 cm into a patient. Panel (a) shows the dose deposited in surrounding water per radiation-induced ionisation as a function of radius from the centre of the agent, i.e. the average radial dose for a single ionisation in the nanoparticle. Panel (b) shows the same data but plotted to show the dose per primary photon incident on the system, i.e. the average radial dose for a single primary photon. The error bars show the standard error in the calculations, determined from at least 7 statistically separate simulations in each case.

Radial distributions for isolated nanoagents are shown in Fig. 2. When viewed on a per-ionisation basis, the RDDs for the three nanoagents look similar, displaying monotonically decreasing energy deposition as the distance from the nanoparticle increases. The ‘waviness’ in these RDDs is related to the characteristic energies of the groups of Auger electrons produced^11^ and their corresponding penetrations^12^. The smaller nanoagents’ RDDs extend to lower radii and correspondingly show larger dose depositions near their surface, even when viewed on a dose-per-primary basis.

### Clusters of nanoparticles

Many copies of each nanoagent were considered to be randomly packed into a 1 µm diameter spherical cluster with a volumetric filling factor of 10%. The local energy enhancement, $L(\overrightarrow{r})$, (i.e. the ratio of the energy deposited due to the nanoparticles to that which would be deposited if no nanoparticles were present) was calculated as a function of three spatial coordinates, denoted by $\overrightarrow{r}$.

From $L(\overrightarrow{r})$, *S*_1_(*r*) was calculated:

1 $${S}_{1}(r)={\int }_{S}L(\overrightarrow{r})\,dA,$$

Similarly, *S*_2_(*r*) was calculated as

2 $${S}_{2}(r)={\int }_{S}{L}^{2}(\overrightarrow{r})\,dA.$$

**Figure 3:**
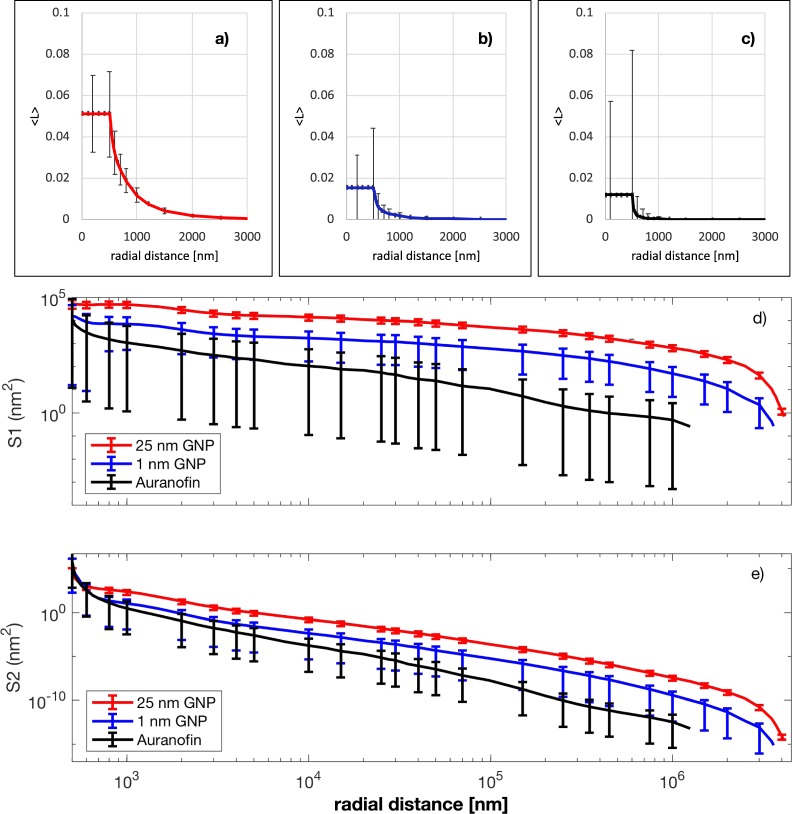
Average local energy enhancement and energy deposition in and around 500 nm radius spherical clusters with a volumetric filling factor of 10%, packed with the three agents under consideration and subjected to a 1 Gy radiation dose. In all plots error bars indicate the standard deviation in the relevant measure taken over 100 different randomly generated clusters, i.e. it is being used to indicate the spread in results expected across a population of such clusters rather than the error in the average obtained. Panels a–c) show the average of the local energy enhancement, 〈*L*〉, as a function of the distance from the centre of clusters made from the three agents. The flat region indicates the average local enhancement inside the cluster but outside of any nanoparticles. Panels (d–e) show the total extra energy deposited into spherical shells *S*_1_(*r*)Δ*r* and *S*_2_(*r*)Δ*r* respectively, centred on the nanoparticle cluster for each of the agents considered.

These functions can be used to evaluate the enhancement to the number of lethal lesions/events for a wide range of scenarios. Furthermore, through examination many such clusters, the statistical spread in the effect across a large population of clusters was deduced, see Fig. 3.

As the size of the nanoagent increases so does <*L*> since more of the nanoparticles interact with the radiation (~10/Gy for the 25 nm radius AuNP, ~1/Gy for the 1 nm radius AuNP and ~0.1/Gy for auranofin). The cluster-to-cluster variations in number of activations increase as the size of the nanoagent decreases - the decreasing number of activation events within the clusters of the smaller nanoagents leads to greater statistical variation across an ensemble. This effect is particularly pronounced inside a cluster, where activation of a single nanoparticle can give rise to a very big change in <*L>*, hence the particular large standard deviations observed for r < 500 nm.

The slow fall-off of *S*_1_(*r*) shows the importance of taking the RDD calculations out to large radii – although the RDD is rapidly falling off, this is largely due to the *r*^−2^ factor as the emitted electrons spread out over 4*π* steradians. This factor is negated when the total dose deposited in a spherical shell is considered.

### Local effect model and effect on cells

Within the framework of the local effect model^13^, the average number of lethal lesions induced can be written as:

$$\langle {N}_{total}\rangle =\alpha D+\beta {D}^{2}+(\alpha D{I}_{1}+\beta D{I}_{2}+2\beta {D}^{2}{I}_{1})/{V}_{sens},$$

where *α* and *β* are the usual parameters of the linear quadratic model, *D* is the dose and *V*_*sens*_ is the sensitive volume. *I*_1_ and *I*_2_ are given by:

3 $${I}_{1}=\int L(\overrightarrow{r})\,dV\approx \int {S}_{1}(r)\frac{{\rm{\Omega }}(r)}{4\pi }dr,$$

4 $${I}_{2}=\int L{(\overrightarrow{r})}^{2}dV\approx \int {S}_{2}(r)\frac{{\rm{\Omega }}(r)}{4\pi }dr.$$

Here Ω(*r*) is a function describing the solid angle of a sphere of radius *r* centred on the nanoparticle cluster and lying inside the sensitive volume. Neglecting the effect of the angular variables implicit in these volume integrals is justified because the ensemble of clusters has no special orientational relationship with respect to either the incoming radiation beam or the biologically sensitive target.

**Figure 4:**
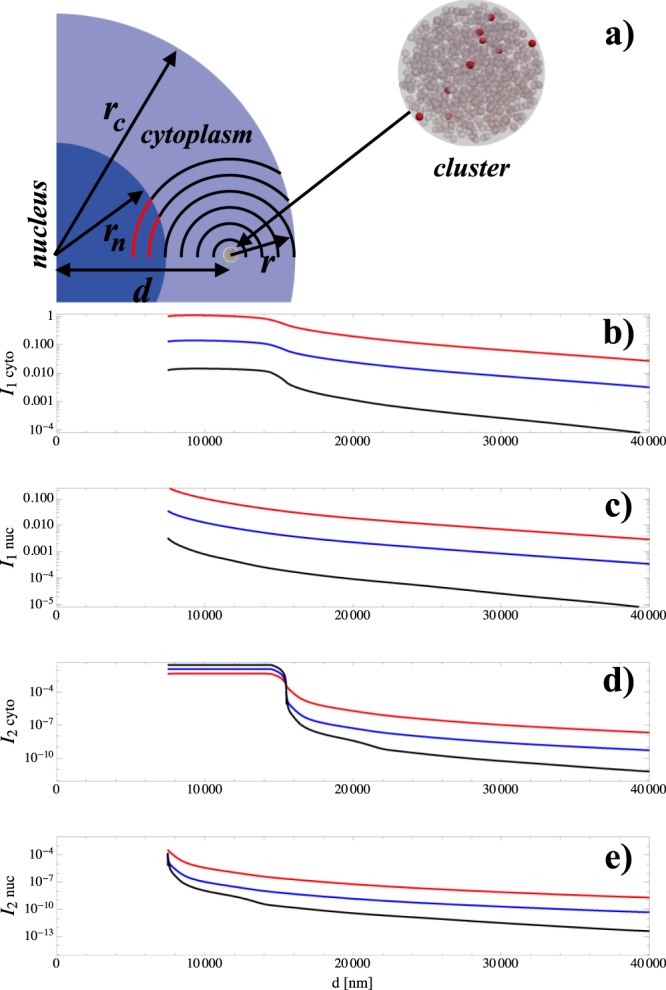
Calculation of the integrals *I*_1_ and *I*_2_ for a model spherical cell. (**a**) Illustration of the geometry under consideration with a 7 μm nuclear radius (*r*_*n*_) and a 15 μm cell radius (*r*_*c*_). A single cluster is ‘injected’ into the cell at a distance *d* from the centre of the cell. For a given distance *r* from the cluster, there is an arc of a circle which lies inside the nucleus (shown in red) and another insider the cytoplasm (shown in black). The solid angle created by rotating each of these arcs about the x-axis gives Ω(*r*) for the nucleus and cytoplasm respectively. Clearly Ω(*r*) is a function of *d* but is independent of the entities loading the cluster. More details of the functional form of Ω(*r*) are given in Supplementary Information. (**b**–**e**) show values of the integrals for both choices of critical volume, for clusters comprised of each of the nanoagents. All of these values have been normalised to the value of *I*_1_ when the cluster formed of 25 nm nanoparticles is just at the edge of the nucleus when the whole cell excluding the cluster is the critical volume, i.e. everything is normalised with respect to the 25 nm nanoparticles’ cluster.

These spherical clusters and cells provide a useful prototypic model with which to compare the number of lethal lesions/events of different nanoagents and different placements of the clusters in the cell. There is some ambiguity over what the biologically sensitive target is for cell killing, i.e. what exactly constitutes *V*_*sens*_. It is generally considered to be the cell nucleus although DNA damage and cell killing are not well correlated when AuNPs were used as dose enhancing agents^14^. In order to examine the effect of different choices for *V*_*sens*_, the nucleus and the cytoplasm were separately considered. Since these clusters of nanoparticles are not generally found inside the cell nucleus^15,16^ only clusters outside the nucleus were considered. Clusters from outside the cell were considered as these could be in a separate cell. These scenarios and the values they produce for *I*_1_ and *I*_2_ are illustrated in Fig. 4.

## Discussion

There is an open question as to whether the volume within the cluster but not occupied by nanoparticles can give rise to biological effect. In the main body of this paper, we consider this not to be the case. However, we discuss the alternative scenario in Supplementary Information. The results shown in Fig. 4 provide insight into achieving optimum physical dose enhancement with clusters of nanoagents. *I*_1_ is far bigger than *I*_2_, allowing *I*_2_ to be neglected in general^17^. Then, the dose enhancement factor (DEF) can be written

5 $$DEF={I}_{1}\frac{(\alpha +2\beta D)}{{V}_{sens}(\alpha +\beta D)}.$$

Apart from *I*_1_, all of the factors on the right-hand side of this equation are dictated by the radiation biology pertaining to the particular clinical scenario – the only factor here which can be influenced for a therapeutic nanoagent is *I*_1_. For both choices of critical target, the curves of *I*_1_ for the different nanoagents are roughly parallel when seen on a logarithmic scale. This means that the associated DEFs will be in a roughly constant ratio, regardless of the placement of the clusters (comparing like-placement to like-placement). The departure from this parallel trend in Auranofin at larger values of *d* is due to the more rapid fall-off in its RDD (Fig. 2) which is a manifestation of the fact that a considerable fraction of the electrons produced by ionizing this molecule are from low-atomic number species.

As is illustrated by Table 2, across all values of *d* the ratio of *I*_1_ for the 25 nm and 1 nm AuNPs is close to the ratio of the number of gold atoms (about 0.14) packed into the clusters. Comparing the 25 nm AuNP to auranofin at the same packing volume, the ratio of numbers of gold atoms is about 1.5 × 10^−2^. This value is close to the ratio of the integrals when most of the contribution comes from near the cluster. However, the value falls away from this value for larger values of *d*, a manifestation of the more rapid fall-off of auranofin’s RDD. This deviation is of little clinical relevance however since it concerns scenarios likely to lead to little clinical enhancement. These findings lead to the suggestion that the major factor in designing nanoagents for dose enhancement is their ability to deliver many high atomic number atoms within the clusters they form, or more generally, within the cell. This is because the extra ionisation events thus created far outweighs the effect of intra-nanoparticle energy attenuation.

**Table 2:** Relative values for the energy deposition integrals *I*_1_ and *I*_2_ for clusters of the three nanoagents, situated at various distances (*d*) from the centre of the cell. The columns headed ‘ratio’ show the ratio of *I*_1_ for the 25 nm AuNPs to *I*_1_ for the nanoagent under consideration.

	nucleus							
	25 nm GNP		1 nm GNP			Auranofin		
d [nm]	*I*_1_	*I*_2_	*I*_1_	*I*_2_	ratio	*I*_1_	*I*_2_	ratio
7,500	0.273	3.91E-4	0.035	1.67E-4	0.13	3.27E-3	1.51E-4	1.2E-2
11,000	0.080	2.03E-6	9.73E-3	5.51E-8	0.12	5.79E-4	5.26E-9	7.3E-3
14,500	0.039	3.34E-7	4.77E-3	8.53E-9	0.12	2.27E-4	2.78E-10	5.6E-3
26,000	0.010	1.59E-8	1.23E-3	3.92E-10	0.12	4.35E-5	8.26E-12	4.3E-3
	**cytoplasm**							
	**25 nm GNP**		**1 nm GNP**			**Auranofin**		
	***I***_**1**_	***I***_**2**_	***I***_**1**_	***I***_**2**_	**ratio**	***I***_**1**_	***I***_**2**_	**ratio**
7,500	1.000	4.69E-3	0.129	0.013	0.13	0.013	0.030	1.3E-2
11,000	1.048	5.07E-3	0.137	0.013	0.13	0.014	0.030	1.3E-2
14,500	0.740	4.61E-3	0.097	0.013	0.13	0.010	0.030	1.4E-2
26,000	0.095	2.61E-7	0.012	6.47E-9	0.12	4.29E-4	1.62E-10	4.5E-3

This result follows directly from the fact that the 25 nm nanoparticle with a 1 nm coating has a higher fractional amount of gold than the 1 nm nanoparticle with a 1 nm coating. Since the total enhancement per cell is given by the enhancement per cluster multiplied by the number of clusters per cell (assuming like-for-like placements) the conclusion is that one would need roughly seven times as many clusters of 1 nm the nanoparticles to penetrate the cell as clusters composed of the 25 nm nanoparticles to have the same effect.

The consideration of coatings is clearly important. If coatings were neglected then the enhancement per cluster would be the same for a given loading fraction, regardless of nanoparticle size.

When comparing dose deposition outside of a cluster of nanoparticles to dose deposition outside of a single, isolated nanoparticle, it is clear that the lower energy Auger electrons provide a smaller contribution in the former case. Due to their small penetration, only a few of these low energy Auger electrons leave the cluster, these being associated with nanoparticles situated near the edge of the cluster. The diminished role of these low energy Auger electrons acts to negate the apparent benefit of the very large dose per ionization seen for the smaller nanoobjects in Fig. 2. This factor, in turn, means that typically heavy atoms near the centre of a large nanoparticle make a similar contribution to the dose deposition outside of the cluster to that made by a heavy atom nearer to the surface – i.e. almost all heavy atoms make an approximately equal contribution (in an average sense, practically for a single scenario it is very few heavy atoms which contribute). This result leads to the finding that the dominant factor in determining factor for physical radiation enhancement effects of heavy-atom bearing nanoparticles is the total number of heavy atoms packed into the cluster under clinically relevant conditions.

Given these factors, it is interesting to make a comparison between a spherical cluster of nanoparticles and a single homogenous sphere composed of elements in the same average proportions and the same total density as the cluster, i.e. to see what difference the fact that the cluster is composed of a nanoparticle + water inhomogeneous mixture rather than an equivalent homogenous sphere. The results of such a comparison are shown in Supplementary Figure S5. Broadly the two representations product the similar results. However, the inhomogeneous representation analysed through the super-position approach we have used has a number of benefits.

The inhomogeneous representation used with the superposition approach gives information about dose heterogeneity near the cluster as is seen in Fig. 1. Furthermore, it can provide more than just information about average enhancements when considering a population of similar clusters as shown by the error bars in Fig. 3. It also provides a better representation of the fall-off of gold at the edge of the cluster, under the assumption that there is a simple bounding surface (spherical in the current case). This is because there is a small fall off in average gold density near the edge of the cluster, the average being over many clusters with the same nanoparticle density and cluster boundary. The fall-off occurs because no nanoparticle centre can lie closer than the nanoparticle radius, denoted by *r*_*np*_, to the edge of the cluster. That is to say the centres of the nanoparticles are all inside a sphere of radius ${r}_{c}-{r}_{np}$, leading to a slight depression of average gold density near the surface of the cluster. This is a contributory factor to the slightly larger value of *I*_1_ observed in Figure S5 for the homogenous case. Another contributory factor is the representation of the stopping power by a constant for the inhomogenous case.

Of course, there are many simplifying assumptions being used to make these clusters of nanoparticles, such as that they pack with constant density, the coatings are rigid etc. One benefit of the inhomogeneous approach using superposition is that it allows these assumptions to be explored readily, something we anticipate will happen in future studies.

Although there is no definitive data set against which the result presented can be compared experimentally, there is plenty of corroborative evidence for the trend observed. Clonogenic survival studies have many potential confounding factors for instance, a poorly defined sensitive volume, so the discussion here is limited to observation of DNA double strand break (DSB) formation. McQuaid *et al*. saw significant enhancement to DNA damage with 1.9 nm nanoparticles^14^ using very low X-ray energies. However, their modelling of the system suggests the effect will vanish at higher energies for these small nanoparticles, whilst it could be expected to persist for the larger ones. The conclusion from their model is corroborated by Jain *et al*.^18,19^ using 160 kVp photons when looking at both DNA damage and repair. Whereas, using 6 MeV clinical beams (which should make the observation of DSB enhancement harder) several reports of DSB enhancement have been presented: Chithrani *et al*. (50 nm diameter)^18^, Bebeco *et al*. (50 nm diameter)^20^ and Zhu *et al*. (20–74 nm)^21^. These observations are broadly in keeping with the trend shown in Fig. 4c.

## Conclusions

A multiscale formalism, underpinned by local effect concepts, has been developed and used to describe the radiation enhancement effects due to clinically realistic clusters of nanoparticles. Using a simplified geometry of spherical cells containing spherical clusters of nanoparticles and considering thousands of such scenarios, sometimes containing millions of nanoparticles it has been possible to draw quite general conclusions relevant to the development of clinical radiation nanomedicines. The results indicate that the large dose enhancements found near small nanoparticles confer no practical advantage and is outweighed by their less efficient packing, unless there is a demonstrable and significant biological effect arising from energy deposition within the cluster. We wish to emphasise that this conclusion is not one which arises from previous studies considering isolated nanoparticles^2–8^ but is a consequence of the proportionality of the DEF to *I*_1_ and the fact that each heavy atom present makes a roughly similar contribution (on a statistically averaged basis) to *I*_1_, due to the fact that the large contributions to the RDD for very small distances have little effect when clinically realistic clusters are considered. Furthermore, this result is not dependent on the subcellular region considered to be sensitive to the effects of radiation, provided the inside the cluster of nanoparticles is not considered to be biologically active.

## Methods

The calculations proceeded through 4 distinct phases. Firstly, a description of all the photons and electrons produced by a clinical linac at a depth of 3 cm into a patient was created. This description took the form of a phase space file which was then remapped to match the size of a single nanoparticle using the technique described by Lin *et al*.^7^. In the second step, the particles (predominantly photons and electrons) from this spectrum were allowed to interact with a single nanoparticle. The outgoing phase space of all particles leaving the nanoparticle after any interaction was recorded, again in a manner similar to that of Lin *et al*. The fraction of particles undergoing interaction was also recorded in this step. In a third step these outgoing particles were transported through water with the dose deposition as a function of radius being recorded. This process gave rise to the RDDs shown in Fig. 2.

Clusters of such nanoagents were constructed stochastically (see Fig. 1 for an example) so that none of the nanoagents overlapped with each other and ensuring they were all bounded by the cluster’s outer radius but otherwise with a uniform probability distribution for each coordinate defining the locations of their centres. The number of interactions between the radiation and each nanoparticle was also determined stochastically for a 1 Gy dose, using the relative interaction probabilities for the nanoagent concerned and a corresponding water nanoparticle (WNP). These parameters were also determined in a manner similar to that of Lin *et al*. In the final step, the RDDs were used as kernel functions from which the dose distribution due to many nanoparticles was determined. This was done using the principle of superposition, with the kernel function being modified to take account of transport through both gold and water inside the clusters. For this step, a set of approximations were used which treated the elements comprising the cluster as a continuous mixture and which neglected the small amount of extra scattering brought about by the heavy atoms in the cluster. Through these approximations, a single RDD kernel could be used to determine the dose at any point due to every nanoparticle in a cluster undergoing an interaction with the incoming radiation beam. In this manner, thousands of calculations, each involving millions of nanoparticles in different clusters became tractable.

Monte Carlo radiation transport simulations were implemented in this work through the Tool for Particle Simulation (TOPAS) software^22^ version 3.1.1, which wraps the Geant4 toolkit^23^ version 10.03.p01. An extension in TOPAS has been implemented to simulate the Auger cascade process^24^, which is the state of the art for the simulation of the full Auger cascade produced by atomic de-excitation. Table S1 in Supplementary Information shows the physics lists used in all the stages of the calculations.

In the first stage, a linac spectrum of 6 MeV TrueBeam Varian linac with a 10 cm^2^ square field from the IAEA nuclear database^25^ was used to irradiate a water phantom of 20 by 20 by 40 cm^3^. In order to generate sufficient statistics, every entry in the database was used 10 times using the technique of particle recycling^26^. A cylindrical scorer of 2.5 cm radius was placed at 3 cm depth in the phantom to score all types of particles traversing its surface as is shown in Figure S1a) in Supplementary Information. A phase space file was constructed recording all of these particle crossings. The x- and y-coordinates of this phase space file was then re-scaled to match the cross-sectional area of the gold or Auranofin nanoagents. Furthermore, the cosine directions of the particles were modified to be parallel to the primary beam, also their weights were modified in order to take into account the difference in area between the phase spaces files in the macro- and the nano-scale, as is explained in^7^.

The particles in those modified phase space files were used to irradiate 1 and 25 nm radius gold nanoparticles, each with a one nanometer citrate coating as is shown in Fig. 1 as well as the Auranofin molecule, which was modelled here as a nano-object of 1 nm radius and density of 1.7 g/cc (the ratio of the sum of the atomic weights to the sum of the atomic volumes). This nano-object was constructed to be contain the elements found in auranofin in their correct proportions as determined from standard atomic volumes^27^. The contracted phase space file representing the linac spectrum at 3 cm depth was recycled 4, 40 and 100 times for the irradiation of 25 and 1 nm GNPs and Auranofin respectively.

The Penelope physics list in Geant4 was used to transport particles inside the nano-objects. The Penelope physics list has been widely applied for nanodosimetry^6,26,28^ due to its high efficiency for the tracking of particles down to 100 eV in materials with Z <99^29^. In this stage, all physics interactions between irradiation and a single GNP were simulated, including the de-excitation processes that involves Fluorescence and the Auger cascades produced in the nanoparticle. All the secondary particles created inside the nanoparticle that were able to escape from it were recorded in a phase space file in ASCII format, which contained the energies, positions, momentum directions and weights of the particles, for subsequent use in the transport simulation used to determine the RDDs.

The Geant4-DNA physics list^30^ was used to transport the electrons from the previous stage inside the water phantom. This physics list is able to track very low energy electrons in water down to a few 7 eV and has been widely validated for the tracking of particles at the nanoscale in water^31,32^. In this stage, all the electrons of the phase space file from the previous stage were released in a 1 cm radius spherical water phantom of as is shown in Figure S2c), the phantom was divided into radial bins of 1 nm from 0 to 300 nm, 97 nm bins from 300 nm to 10 um, 1 um bins from 10 um to 100 um, and 110 um bins from 100 um to 1 cm, in order to calculate the Radial Dose distribution (RDD).

Two mathematically equivalent approaches were used, depending on the probability of a single nanoagent having more than one interaction with the radiation. When this probability was not negligible (25 nm radius AuNPs only in this study) all the AuNPs in the cluster were generated, ensuring the entire volume of each AuNP was inside the cluster and that none of them overlapped. For each nanoparticle, a number of ionisation events was generated stochastically using the Poisson probability distribution function with the mean number of events from the ionisation rate determined from the single nanoparticle simulations for AuNP and a WNP of the same size.

This set was then pruned by discarding all those with zero ionization events. When the double-interaction probability was negligible (1 nm radius and Auranofin), only single ionization events need to be considered. This was done by generating the number of nanoobjects undergoing interaction (binomial probability distribution function, mean from the ionisation rate determined from the single nanoparticle simulations) and their locations.

The means to calculate the dose-deposition draws conceptually from the undergraduate physics problem of calculating the electric potential charges using the principle of superposition of charges. Accordingly, we borrow relevant terminology from the subject of electrostatics, referring to the point where we wish to calculate dose as the ‘field point’ and the centre of the nanoagent currently under consideration (i.e. producing the dose) as the ‘source point’. In this analogy the RDD takes the role of the Coulomb potential of a single charge. Due to the way the RDD is calculated, it already accounts for the attenuation of energy as the radiation passes through the water.

The relevant equation of electrostatics is:

$${V}_{tot}({\mathop{r}\limits^{\rightharpoonup }}_{j})={\sum }_{i}{q}_{i}\frac{1}{4\pi {\varepsilon }_{0}|{\mathop{r}\limits^{\rightharpoonup }}_{i}-{\mathop{r}\limits^{\rightharpoonup }}_{j}|},$$

with the sum running over all charges. By analogy this becomes:

$${D}_{tot}({\mathop{r}\limits^{\rightharpoonup }}_{j})={\sum }_{i}{n}_{i}RDD(|{\mathop{r}\limits^{\rightharpoonup }}_{i}-{\mathop{r}\limits^{\rightharpoonup }}_{j}|),$$

where *n*_*i*_ is the number of ionisation events the *i*^*th*^ nanoparticle has undergone. This result neglects the effect of other nanoparticles on the subsequent transport. There are two components to consider, scattering and energy deposition, as the radiation transports from the nanoparticle to the field point. We simply neglect scattering – this is justified because the system is quasi-spherically symmetric. Furthermore, Monte Carlo transport calculations explicitly considering the effect of a nanoparticle on the electron transport through a cluster (see Figure S3) show this to be a good approximation when scoring total energy deposition in concentric spheres.

Energy lost due to transport through the cluster is accounted for by noticing that the electron particle is the main responsible for the depositing of energy in the medium when the nanoparticle is activated by gamma irradiation. Furthermore, the ratio of the stopping power for a water/gold mixture to the stopping power of water alone is constant to a very good approximation (see Figure S4). This means that the transport of an electron through some distance *d* in a water/gold mixture can be considered approximately equivalent to the transport through a distance *k* × *d* where *k* is the (asymptotic) ratio of the stopping powers shown in Figure S4.

Making this approximation, the superposition equation becomes:

$${D}_{tot}({\mathop{r}\limits^{\rightharpoonup }}_{j})={\sum }_{i}{n}_{i}RDD(g(|{\mathop{r}\limits^{\rightharpoonup }}_{i}-{\mathop{r}\limits^{\rightharpoonup }}_{j}|)),$$

where $g(|{\mathop{r}\limits^{\rightharpoonup }}_{i}-{\mathop{r}\limits^{\rightharpoonup }}_{j}|)$ is a scalar function accounting for the fraction of the trajectory which passes through the gold/water mixture. Effectively *g*(*r*) maps from the distance between source point and field point to a new (slightly further) distance thereby accounting for the transport through the gold/water mixture in an average sense (i.e. the gold density is an approximate representation of the average density of gold over all of the clusters which could have given rise to the specific configuration of ionised nanoparticles). Doing the calculation for ${D}_{tot}({\mathop{r}\limits^{\rightharpoonup }}_{j})$ for a *n*_*i*_ set to the value for a 1 Gy fraction (to water) gives $L(\overrightarrow{r})$, (i.e. the ratio of the energy deposited due to the nanoparticles to that which would be deposited if no nanoparticles were present).

*g*(*r*) was determined geometrically by taking a straight line from the source point to the field point and performing the following sum:

$$g(r)={r}_{np}+k\,{l}_{in}+{l}_{out},$$

where *r*_*np*_ is the radius of the nanoparticle, *l*_*in*_ is the distance travelled through the cluster but outside of the source nanoparticle but still inside the cluster and *l*_*out*_ is the remaining distance to the source point. This formalism assumes a uniform probability density for finding gold anywhere inside the cluster but outside of the source nanoparticle. Strictly this is incorrect as the exclusion of nanoparticle centres within 2*r*_*np*_ of each other or within *r*_*np*_ of the edge of the cluster produces a slight suppression of probability in some regions of a given cluster, resulting in a small commensurate increase elsewhere. This approximation can be removed by taking the line integral of the ratio of stopping powers along the trajectory represented by *l*_*in*_. However, this more exact approach is computationally much more expensive and numerical simulation suggested the effect is negligible.

*S*_1_(*r*) and *S*_2_(*r*) were calculated by numerical integration of equations 1 and 2 respectively, the domain of integration being the surface of a sphere of radius *r* centred on the cluster. This was achieved by generating 1000 points on the surface of the sphere using Vogel’s method^33^, finding $L(\overrightarrow{r})$ and ${L}^{2}(\overrightarrow{r})$ using the the energy deposition at these points and then multiplying them by the area of the sphere. These functions (shown in Fig. 3) were stored for future use.

Due to the symmetry of the prototypical cells being simulated, the integral functions *I*_1_ and *I*_2_ can be calculated numerically from *S*_1_(*r*) and *S*_2_(*r*) provided Ω(*r*) is known. This function can be deduced analytically for either the cell nucleus or the cytoplasm from simple geometrical considerations. These analytic representations of Ω(*r*) were used along with interpolated representations of *S*_1_(*r*) and *S*_2_(*r*) to numerically determine the values of the integrals *I*_1_ and *I*_2_ shown in Fig. 4.

Computer codes used to perform these calculations will be made publicly available through the University of Manchester’s institutional repository upon its acceptance for publication.

## Supplementary information

Supplementary Information

## References

[1] Bonvalot S (2017). First-in-Human Study Testing a New Radioenhancer Using Nanoparticles (NBTXR3) Activated by Radiation Therapy in Patients with Locally Advanced Soft Tissue Sarcomas. Clin Cancer Res.

[2] McMahon SJ (2011). Biological consequences of nanoscale energy deposition near irradiated heavy atom nanoparticles. Sci. Rep..

[3] McMahon SJ (2011). Nanodosimetric effects of gold nanoparticles in megavoltage radiation therapy. Radiother. Oncol..

[4] Lechtman E, Pignol JP (2017). Interplay between the gold nanoparticle sub-cellular localization, size, and the photon energy for radiosensitization. Sci. Rep..

[5] Wälzlein C, Scifoni E, Krämer M, Durante M (2014). Simulations of dose enhancement for heavy atom nanoparticles irradiated by protons. Phys. Med. Biol..

[6] Sung W (2017). Dependence of gold nanoparticle radiosensitization on cell geometry. Nanoscale.

[7] Lin Y, McMahon SJ, Scarpelli M, Paganetti H, Schuemann J (2014). Comparing gold nano-particle enhanced radiotherapy with protons, megavoltage photons and kilovoltage photons: a Monte Carlo simulation. Phys. Med. Biol..

[8] Lin Y, McMahon SJ, Paganetti H, Schuemann J (2015). Biological modeling of gold nanoparticle enhanced radiotherapy for proton therapy. Phys. Med. Biol..

[9] McMahon SJ, McNamara AL, Schuemann J, Prise KM, Paganetti H (2016). Mitochondria as a target for radiosensitisation by gold nanoparticles. Journal of Physics: Conference Series.

[10] Zygmanski P, Sajo E (2016). Nanoscale radiation transport and clinical beam modeling for gold nanoparticle dose enhanced radiotherapy (GNPT) using X-rays. The Br. J. Radiol..

[11] Currell, F. & Villagomez-Bernabe, B. Physical and Chemical Processes for Gold Nanoparticles and Ionizing Radiation in Medical Contexts Ch 15. In Gold Nanoparticles for Physics, Chemistry and Biology (Second Edition) eds Louis, C. & Pluchery, O. (World Scientific, London 2017, isbn 1786341247).

[12] Meesungnoen J, Jay-Gerin JP, Filali-Mouhim A, Mankhetkorn S (2002). Low-energy electron penetration range in liquid water. Radiat. Res..

[13] Scholz M, Kraft G (1996). Track structure and the calculation of biological effects of heavy charged particles. Advances in Space Research.

[14] McQuaid HN (2016). Imaging and radiation effects of gold nanoparticles in tumour cells. Sci. Rep..

[15] Coulter JA (2012). Cell type-dependent uptake, localization, and cytotoxicity of 1.9 nm gold nanoparticles. International J. Nanomedicine.

[16] Botchway SW, Coulter JA, Currell FJ (2015). Imaging intracellular and systemic *in vivo* gold nanoparticles to enhance radiotherapy. The Br. J. Radiol..

[17] Brown JM, Currell FJ (2017). A local effect model-based interpolation framework for experimental nanoparticle radiosensitisation data. Cancer nanotechnology.

[18] Jain S (2011). Cell-specific radiosensitization by gold nanoparticles at megavoltage radiation energies. Int. J. Radiat. Oncol. Biol. Phys..

[19] Chithrani DB (2010). Gold nanoparticles as radiation sensitizers in cancer therapy. Radiat. Res..

[20] Berbeco RI (2012). DNA damage enhancement from gold nanoparticles for clinical MV photon beams. Radiat. Res..

[21] Zhu CD (2015). Synthesis of novel galactose functionalized gold nanoparticles and its radiosensitizing mechanism. J. Nanobiotechnol.

[22] Perl, J., Shin, J., Schümann, J., Faddegon, B. & Paganetti, H. TOPAS: an innovative proton Monte Carlo platform for research and clinical applications. *Med. Phys*. **39** (2012). 10.1118/1.4758060 PMC3493036 23127075

[23] Agostinelli, S. *et al*. Geant4-A simulation toolkit. *Nuclear Instruments and Methods A* **506** (2003).

[24] Incerti S., Suerfu B., Xu J., Ivantchenko V., Mantero A., Brown J.M.C., Bernal M.A., Francis Z., Karamitros M., Tran H.N. (2016). Simulation of Auger electron emission from nanometer-size gold targets using the Geant4 Monte Carlo simulation toolkit. Nuclear Instruments and Methods in Physics Research Section B: Beam Interactions with Materials and Atoms.

[25] Online: https://www-nds.iaea.org/phsp/photon/Varian_TrueBeam_6MV/. Accessed on the 30^th^ of April 2018.

[26] Mayles, P., Nahum, A. & Rosenwald, J. C. *Handbook of Radiotherapy physics –Theory and Practice*, ISBN: 13:978-1-4200-1202-6 (Taylor and Francis Group, 2007)

[27] Online: https://www.colorado.edu/engineering/MCEN/MCEN2024/02_Atomic%20Volume.html. Accessed on the 30^th^ of April 2018.

[28] Lechtman E, Chattopadhyay N, Cai Z, Mashouf S, Reilly R, Pignol J P (2011). Implications on clinical scenario of gold nanoparticle radiosensitization in regards to photon energy, nanoparticle size, concentration and location. Physics in Medicine and Biology.

[29] Salvat, F., Fernández-Varea, J. M., Acosta, E. & Sempau, J. OECD/NEA Data Bank. *OECD Publications* (2001).

[30] Bernal M.A., Bordage M.C., Brown J.M.C., Davídková M., Delage E., El Bitar Z., Enger S.A., Francis Z., Guatelli S., Ivanchenko V.N., Karamitros M., Kyriakou I., Maigne L., Meylan S., Murakami K., Okada S., Payno H., Perrot Y., Petrovic I., Pham Q.T., Ristic-Fira A., Sasaki T., Štěpán V., Tran H.N., Villagrasa C., Incerti S. (2015). Track structure modeling in liquid water: A review of the Geant4-DNA very low energy extension of the Geant4 Monte Carlo simulation toolkit. Physica Medica.

[31] Incerti S., Ivanchenko A., Karamitros M., Mantero A., Moretto P., Tran H. N., Mascialino B., Champion C., Ivanchenko V. N., Bernal M. A., Francis Z., Villagrasa C., Baldacchino G., Guèye P., Capra R., Nieminen P., Zacharatou C. (2010). Comparison of GEANT4 very low energy cross section models with experimental data in water. Medical Physics.

[32] Incerti, S., Douglass, M., Penfold, S., Guatelli, S. & Bezak, E. Review of Geant4-DNA applications for micro and nanoscale simulations. *Phys. Med*. **32** (2016). 10.1016/j.ejmp.2016.09.007 27659007

[33] Vogel Helmut (1979). A better way to construct the sunflower head. Mathematical Biosciences.

