# Supplementary material for: Physical Radiation Enhancement Effects Around Clinically Relevant Clusters of Nanoagents in Biological Systems
Source: Sci Rep. 2019 May 31;9:8156. doi: 10.1038/s41598-019-44482-y (PMC6544818; doi:10.1038/s41598-019-44482-y)
Supplement: Supplementary file 1 — Supplementary Information [file 41598_2019_44482_MOESM1_ESM.pdf]

Supplementary Information

Physical Radiation Enhancement Effects Around Clinically Relevant Clusters of Nanoagents in Biological Systems.

B. Villagomez-Bernabe[1] and F. J. Currell[2,*]

[1.]School of Mathematics and Physics, Queen's University, Belfast BT7 1NN, UK

[2.] The Dalton Cumbrian Facility and the School of Chemistry, The University of Manchester, Westlakes Science & Technology Park, Moor Row, Cumbria, CA24 3HA

frederick.currell@manchester.ac.uk

S1. Details of the Calculation

Stage	Material	Physics List
1	Water phantom at macro scale	Penelope
2	GNPs and Auranofin molecule	Penelope
3	Water phantom at nano/micro scale	Geant4-DNA

Table S1. Physics lists used for each stages of the Monte Carlo simulations. The production cuts for the simulation at the macroscale (i.e., stages 1, transport of particles in the water phantom) was set to 0.1 um and 1 nm at nanoscale (stages 2 and 3 irradiation of the GNP). A note has been added to this effect.

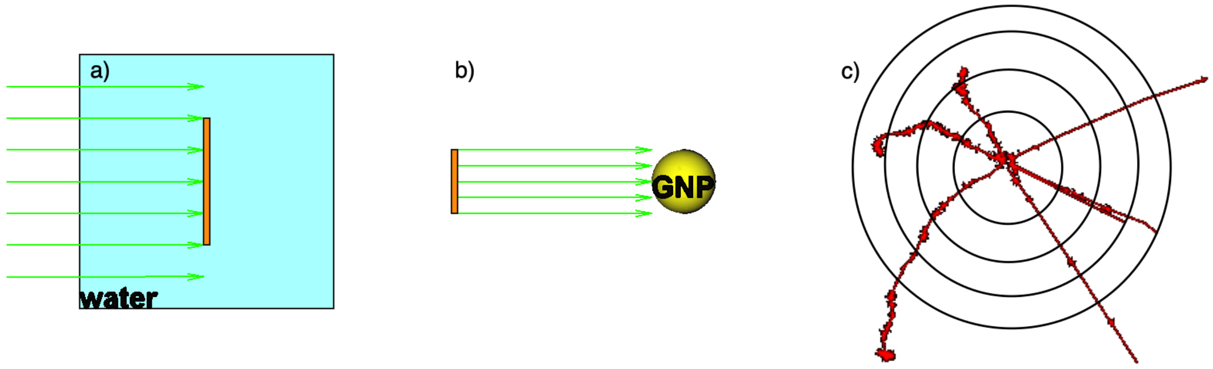

Figure S2. a) a water phantom of 20*20*40 cm3 is irradiated with a 6 MeV TrueBeam Varian linac and the particles were scored on a plane at 3 cm depth (orange plane). b) The field size in the phase space was shrunk to irradiate the nanoparticle. c) Tracking of electrons in water at the nanoscale.

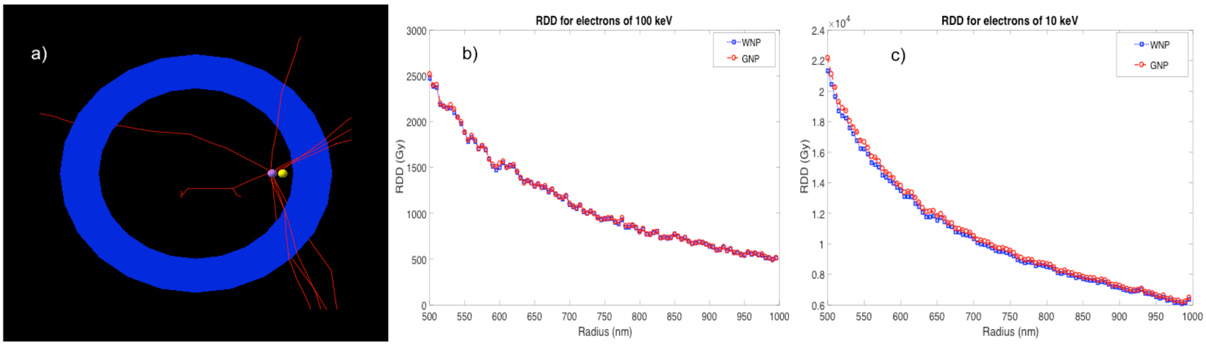

Figure S3: Illustration of the effect of nanoparticle scattering on electron transport through the cluster. An isotropic spherical source of monoenergetic electrons of radius 25 nm was placed 392 nm from the centre of a large spherical volume of water as illustrated in panel a). This source then acted to model electrons a portion of the electrons emitted from an irradiated nanoparticle. A 25 nm radius nanoparticle of either gold or water placed with

its centre a further 54 nm from the centre of this volume (i.e. with its surface 4 nm from the source). The resultant radial dose for transport of 100,000 electrons deposited in spherical shell, from 500 nm outwards from the centre of the simulation were recorded. The results for 100 and 10 keV electrons with a gold nanoparticle (GNP) and a water nanoparticle (WNP) are shown in panels b) – c) respectively. The similarity between the two dose distributions in each case indicates that electron-scattering by nanoparticles within the cluster has a very small effect.

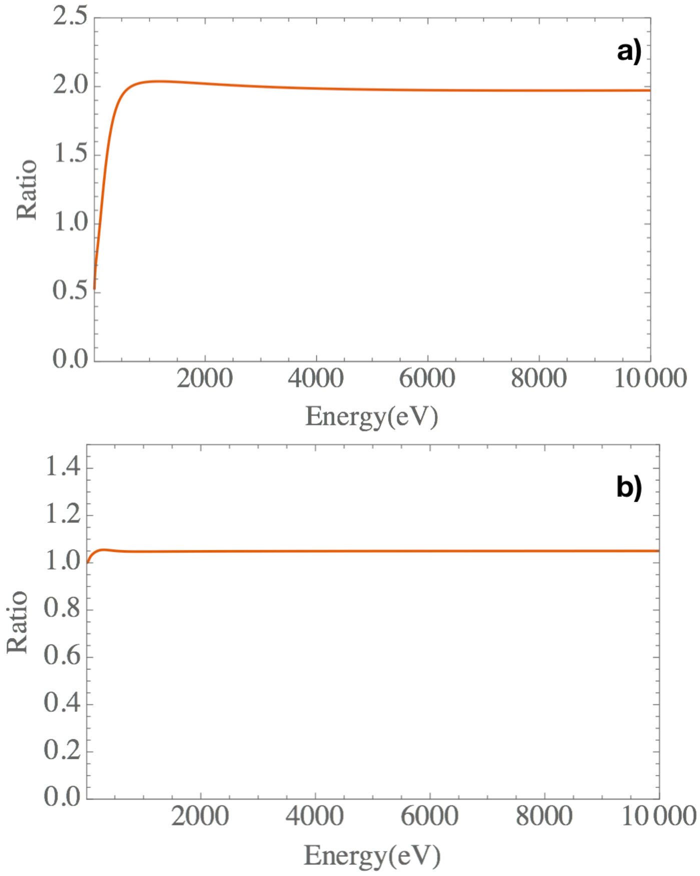

Figure S4: Ratio of the electron stopping power for a prescribed mixture of elements, to that of water as a function of electron energy, a) for a mixture with fractional densities of each atomic species selected present in a 10%: 90% by volume mixture of the 25 nm radius citrate capped gold nanoparticles:water and b) the same for a 10%:90% auranofin:water

mixture. The formalism for calculating the electron stopping powers of mixtures was taken from [1].

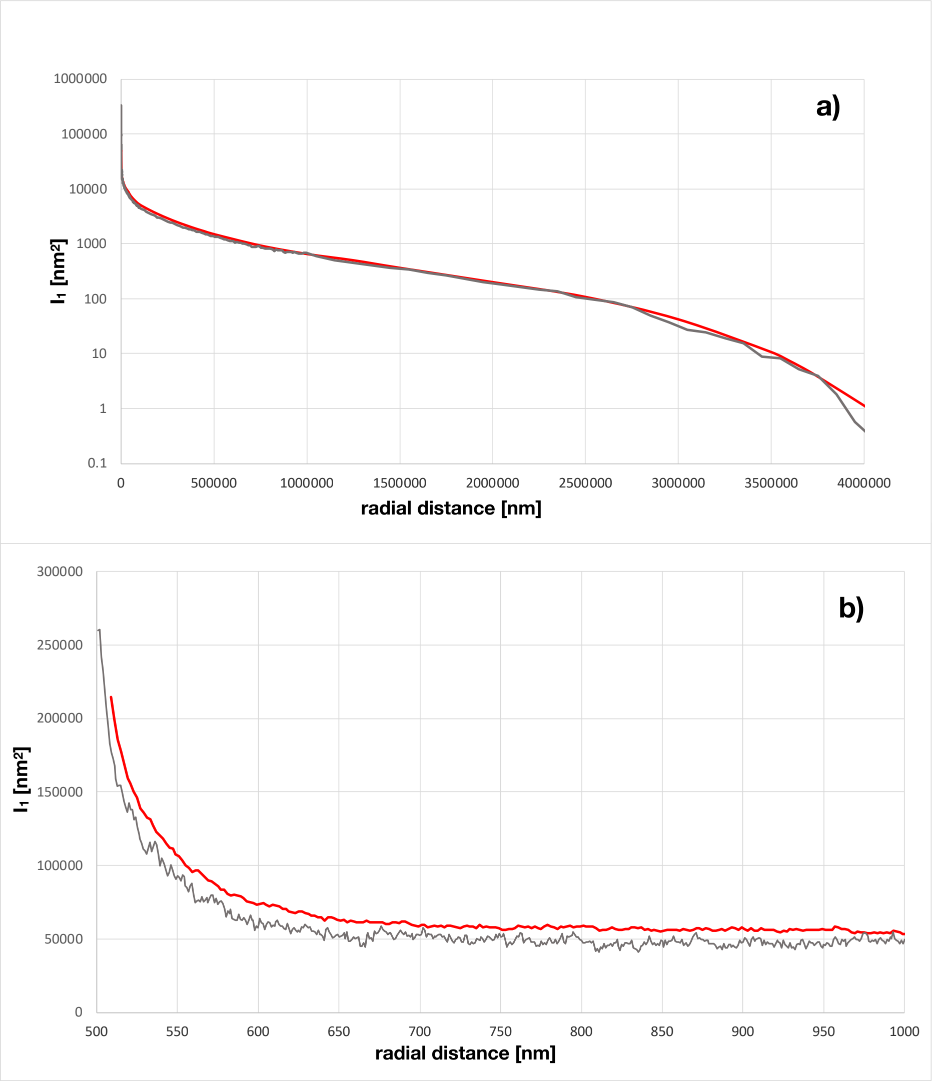

Figure S5. Comparison of S1 calculated for a discrete cluster of 25 nm radius AuNPs (red lines) compared to a single sphere containing a homogenous mixture of elements with corresponding fractional densities (grey lines). Panel a) shows the two representations give broadly similar results whilst panel b) shows that close to the surface of the cluster there is a small but significant difference as is discussed in the main text.

S2. The functional form of Ω(r)

Applying the cosine rule, one can readily determine the angles subtended by the red and black arcs shown in figure 1a as a function of the parameters r_n , r_c, r, and d. These angles can then be used to determine the solid angles of the respective spheres inside the cell (nucleus) or cytoplasm (black) through the relationship

$$\Omega = 2\pi(1 - cos\theta).$$

Practically this is done by subtracting the solid angle of the sphere inside the nucleus from that inside the cell to give the solid angle inside the cytoplasm.

S3. Consideration of the inside of the cluster being biologically active:

There is also an open question as to whether the volume within the cluster which is not occupied by nanoparticles can give rise to biological effect. Clearly the volume occupied by nanoparticles cannot be sensitive. However, dose deposited in the remainder of the volume of the cluster could possibly lead to biological activity, for example through the production of genotoxic species which subsequently transport to biological targets. Allowing this possibility, the expressions for I_1 and I_2 would become:

$$I_1 = (\int S_1(r)\frac{\Omega(r)}{4\,\pi}dr + V_c < L_{in} >),$$

$$I_2 = (\int S_2(r)\frac{\Omega(r)}{4\,\pi}dr + V_c < {L^2}_{in} >).$$

Here the extra terms $V_c < L_{in} >$ and $V_c < L^2{}_{in} >$ describe energy deposition in the cluster but outside of the nanoparticles. V_c is the volume of the cluster which is not occupied by nanoparticles and $< L_{in} >$ is the average dose deposited in V_c due to the nanoparticles in the cluster for a unit dose deposited directly by the radiation (i.e. in the absence of nanoparticles) in the same volume. $< L^2{}_{in} >$ is an analogous quantity but with the average of the dose-squared being used. Practically these expressions give upper bounds for I_1 and I_2 since the inside of the cluster is unlikely to be as biologically sensitive as the outside (if at all).

The trend reported where biological effect roughly scales with the number of heavy atoms contained in a cluster, of course, breaks down if the inside of the cluster is considered to be biologically active, due to the large contribution to $V_c < L_{in} >$ or $V_c < L^2{}_{in} >$ from the RDDs very close to the nanoparticles.

25 nm GNP		1 nm GNP		Auranofin	
$V_c < L_{in} >$ 0.149	$V_c < L^2{}_{in} >$0.474	$V_c < L_{in} >$ 0.045	$V_c < L^2{}_{in} >$75.4	$V_c < L_{in} >$ 0.035	$V_c < L^2{}_{in} >$73.4

Table S2: Values of the extra contributions to the integrals I_1 and I_2 for clusters of the three nanoagents if the insides of the clusters were considered to be biologically active.

References (S)

S1. Nguyen-Truong, H. T. Modified Bethe formula for low-energy electron stopping power without fitting parameters *Ultramicroscopy* 149 26-33 (2015)
